# A Competing Nomogram to Predict Survival Outcomes in Invasive Micropapillary Breast Cancer

**DOI:** 10.7150/jca.27955

**Published:** 2019-11-01

**Authors:** Dan Li, Chenhan Zhong, Yi Cheng, Ning Zhu, Yinuo Tan, Lizhen Zhu, Dong Xu, Ying Yuan

**Affiliations:** 1 Department of Medical Oncology, The Second Affiliated Hospital, Zhejiang University School of Medicine, Hangzhou, Zhejiang Province, China.; 2 Department of Surgical Oncology, The Second Affiliated Hospital, Zhejiang University School of Medicine, Hangzhou, Zhejiang Province, China.; 3 Cancer Institute (Key Laboratory of Cancer Prevention and Intervention, Chinese National Ministry of Education; Key Laboratory of Molecular Biology in Medical Sciences, Zhejiang Province, China), The Second Affiliated Hospital of Zhejiang University School of Medicine, Hangzhou, Zhejiang Province, China.

**Keywords:** invasive micropapillary carcinoma, tumor characteristics, survival outcomes, nomogram

## Abstract

**Background:** Although it is widely accepted that invasive micropapillary carcinoma (IMPC) presents more aggressive behavior and has a higher aggressive behavior, the prognosis of IMPC compared with invasive ductal carcinoma (IDC) remains controversial. We conducted this study to explore gene expression profiles of IMPC and establish a competing nomogram that predicts the survival outcomes across these two groups of patients.

**Methods:** Data from the Surveillance, Epidemiology, and End Results (SEER) database were reviewed. Propensity score matching (PSM) was used to adjust for potential baseline confounding between IMPC and IDC group. The Kaplan-Meier method was used to calculate the occurrence of overall mortality. The Gray method was used to estimate the rate of breast cancer specific death (BCSD). A competing regression model was used to evaluate factors associated with BCSD. A nomogram based on the competing risk regression model was established to predict individual outcomes. IMPC-specific gene expression profiles were explored using microarrays data from the Gene Expression Omnibus (GEO) database. Gene ontology (GO) and Kyoto Encyclopedia of Genes and Genomes pathway (KEGG) enrichment analyses were performed.

**Results:** In this study, 330786 (99.62%) patients with IDC 1247 (0.38%) patients with IMPC were included. Patients with IMPC had more lymph node involvement and a larger tumor size compared with those with IDC. After PSM, many distributional differences were eliminated, showing that the IMPC and IDC group were more similar. Patients with IMPC had a favorable prognosis with statistical significance compared with patients with IDC (overall mortality HR = 0.68; 95%CI, 0.53-0.86; *P* = 0.002). Based on Gray method, patients with IMPC had a favorable prognosis with significant statistical significance compared with patients with IDC (BCSD SHR = 0.64; 95%CI, 0.47-0.88; *P* = 0.006). Multivariate analysis based on competing risk model demonstrated that IMPC was a favorable independent factor for BCSD. The nomogram could accurately predict BCSD with a high internal and external validated C-index (0.835, 0.818 respectively). A total of 53 upregulated differentially expressed genes (DEGs) and 40 downregulated DEGs of IMPC was identified. The GO analysis results showed that downregulated DEGs were significantly enriched in extracellular structure organization, extracellular matrix, cell-substrate adhesion junction. KEGG analysis of selective gene sets shows that downregulated DEGs significantly enriched for processes related to carbon metabolism, Rap1 signaling pathway.

**Conclusion:** In the current study, IMPC accounted for 0.38% of the entire cohort. IMPC was found to be a favorable independent prognostic factor. The present study identified gene expression profiles and signal pathways of IMPC. The developed nomogram can help the oncologists to predict individual outcomes more accurately.

## Introduction

Invasive micropapillary carcinoma (IMPC) is an aggressive histological subtype of invasive cancer of the breast 1, 2. Firstly described by Fisher in 1980 3, pure IMPC is characterized by tumor cells arranged in typical nets papillary surrounded by an obliterated lumina 4, 5. The formal concept of IMPC was initially put forward by Siriaunkgul et al in 1993 6. Given its unique morphological characteristics and greater invasiveness, IMPC was listed as an independent subtype in the 2003 World Health Organization classification of breast cancer 7. Although it is widely accepted that IMPC has a more aggressive behavior, it remains unclear whether this behavior translates into poor prognosis 8, 9. What's more, given its dissimilarity to the common histology of breast cancer, the rareness of IMPC should be emphasized in clinical practice 10.

Previous studies on IMPC often had bias caused by a small patient cohort and the limited follow-up time due to tumor rarity. The strategies in the clinical guidelines for the treatment of IMPC are based on those for the treatment of invasive ductal carcinoma (IDC). Therefore, a specific prognosis evaluation system for IMPC should be utilized to guide treatment strategies in clinical practice 1, 11. On other hand, the causes of death other than cancer are competing risks among patients with early breast cancer. Thus the cause-specific endpoints were always chosen as endpoint for these studies. However, the Kaplan-Meier method and the Cox proportional hazards model fail to estimate cancer specific death 12.

More recently, nomogram as a prognostic and predictive model have been proposed as reliable and alternative tools to integrate pathological, molecular and clinical characteristics of a disease and consequently establish an evaluation system for the prediction of individual patient outcomes 13. We attempted to construct a nomogram for IMPC and IDC based on nationwide, population-based clinicopathological and molecular data from Surveillance, Epidemiology, and End Results (SEER) database.

## Patients and methods

### Inclusion and exclusion criteria

The SEER database includes information on cancer samples from 18 population-based cancer registries and is updated annually to reflect the latest real-world information about cancer. The SEER. Stat software was used to identify patients with breast cancer from January 2001 to December 2015. The specific inclusion criteria were identified as follows: (1) histology ICD-O-3 was limited to IDC (8500/3), IMPC (8507/3), (2) patients without distance metastasis at diagnosis, and (3) the age at diagnosis was between 20 and 80. The exclusion criteria were as follows: (1) the detailed information lacks race, marital status or age, (2) patients with multiple primary tumors, (3) survival time less than 1 month, (4) unknown cause of death, (5) patients with metastatic disease, (6) T classification: unknown, and (7) N classification: unknown.

We randomly divided the entire cohort into a train cohort (75%) and validation cohort (25%) for development and validation of the competing risks nomogram.

The gene expression profiles of GSE66418 were downloaded from the Gene Expression Omnibus (GEO) database, which contains 124 samples, including 73 IMPC samples and 51 matched invasive carcinomas of no special type samples (ICNST). GSE66418 was based on the GPL6801platform [HG-U133_Plus_2] Affymetrix Human Genome U133 Plus 2.0 Array, and submitted by Gruel et al 14.

### Variable declaration

Race was divided into white, black and others. The age was regrouped as 20-50yrs (young) and 51-80yrs (old). Marital status was listed as married, single and divorced. The hormone receptor (HR) status of the tumor was stratified as HR-positive and HR-negative. The chemotherapy variable was classified only as chemotherapy “yes” or “no/unknown”.

### Statistical analysis

Propensity score matching (PSM) is a statistical method that reduces confounding from measured variables in observational data. In regression adjustment for the propensity score, a treatment case is matched with one or more control cases based on their propensity scores 15. Patients with the same propensity score have the same distribution of measured confounders. Provided that there is no unmeasured confounding, we obtain unbiased estimates of the treatment effect by comparing treatment groups within levels of the propensity score 16.

Patients with early stage low-risk breast cancer detected by mammography screening programs, are expected to survive breast cancer are at a higher risk of non-BCSD 17. To obtain unbiased estimates of the risk of BCSD, we applied a proportional subdistribution hazards regression (competing risk model), which connects the regression coefficients to a cumulative incidence function to estimate the unbiased risks in the presence of competing risks 18.

Categorical variables were compared using the Chi-Squared test. BCSD was calculated from the date of breast cancer diagnosis to the date of death due to breast cancer. Death due to other causes was defined as competing risks. Overall mortality was analyzed using the Kaplan-Meier method between groups with a log-rank test for significance. A proportional subdistribution hazards regression (competing risk model) was used to determine factors associated with BCSD. Prognostic factors with a *P* value < 0.05 in univariate analysis were incorporated into multivariate analysis. A *P* value < 0.05 was considered to be statistically significant in the multivariate analysis, while subdistribution hazard ratio (SHR) and a 95% confidence interval (CI) were used to adjust for prognostic variables. BCSD was set as the primary endpoint, because one of the primary goals of therapy is to reduce death attributable to the underlying breast cancer. In addition, Overall mortality was set as the secondary endpoint. To avoid multicollinearity, the multivariable analysis included the variables of T-classification, N-classification, ER (Estrogen receptor) status and PR(Progesterone receptor) status rather than the variables of stage and HR status.

A nomogram was established based on the competing risk regression model to predict the individual outcomes of BCSD at 3 years, 5 years, and 10 years. Age at diagnosis, race, marital status, tumor location, differentiation grade, histology, T classification, N classification, and ER and PR status were included in the model. The prognostic accuracy of the nomogram model was validated by internal validation, with bootstrapping to calculate a relatively unbiased measure of the ability to discriminate between patients as measured by the concordance index (C-index). The calibration was evaluated by plotting the observed Gray 3-year proportions, 5-year proportions and 10-year proportions compared with the corresponding nomogram for the 3-year, 5-year or 10-year predicted BCSD. Likewise, an external validation was performed in the validation cohort. The C-index was also used to quantify the discrimination ability of the prediction model. Since this study utilized data from the SEER database, which contained no personal identifying information, ethical approval and informed consent from patients in current study were not required.

All analysis was performed using the statistical software and R software (version 3.5.0). Rms package was used to performance survival analysis. Limma package was used to identify differentially expressed genes (DEGs) between IMPC and ICNT groups^19^. The adjusted *P* values < 0.05 and absolute fold change (FC) ≥ 2.0 were considered as the cutoff values. ClusterProfiler package was used to analyze the functions of DEGs 20. Gene ontology (GO) and Kyoto Encyclopedia of Genes and Genomes pathway (KEGG) enrichment analyses were performed.

## Result

### Clinicopathological characteristics

A total of 332033 patients with early breast cancer were included in the study. The median age of the 330786 (99.62%) patients with IDC was 57 years compared with 58 years in the 1247 (0.38%) patients with IMPC. IMPC patients with larger tumor size (T3) (8.74% vs 4.54%; *P* < 0.001), more lymph node involvement (51.48% vs 33.73%, *P* < 0.001), and a higher stage (stage II 38.97% vs 37.88%, stage III 23.5% vs 12.45%, *P* < 0.001) compared with those with IDC. And IMPC was also related to a higher frequency of ER-positive and PR-positive status (88.69% vs 77.52%, *P* < 0.001; 78.75% vs 67.51%, *P* < 0.001, respectively) as well as an obviously higher frequency of HR-positive status (89.17% vs 78.87%, *P* < 0.001) compared with IDC. Additionally, similar percentages of patients with either IMPC or IDC were receiving. Compared to IDC group, IMPC group has a significant higher rate of receiving radiation treatment (59.42% vs 56.36%, *P* = 0.029) and chemotherapy treatment ((55.73% vs 48.92%, *P* < 0.001). IMPC group had a significant lower rate of breast conserving surgery (56.05% vs. 61.18%, *P* < 0.001). 35.12% of patients had at least 10 lymph nodes removed in IMPC group, which was significant higher than 29.48% in IDC group (*P* < 0.001). Given the clear group differences between IDC and IMPC group, propensity scores describing the likelihood of histology contingent on covariates were used to reweight the patient population for each group. Many distributional differences were eliminated, yielding IDC and IMPC group that were more similar (**Supplementary Figure 1**). The characteristics of the patients were shown in **Table 1**.

### Survival analysis based on the Kaplan-Meier and Gray method

**Figure 1:**
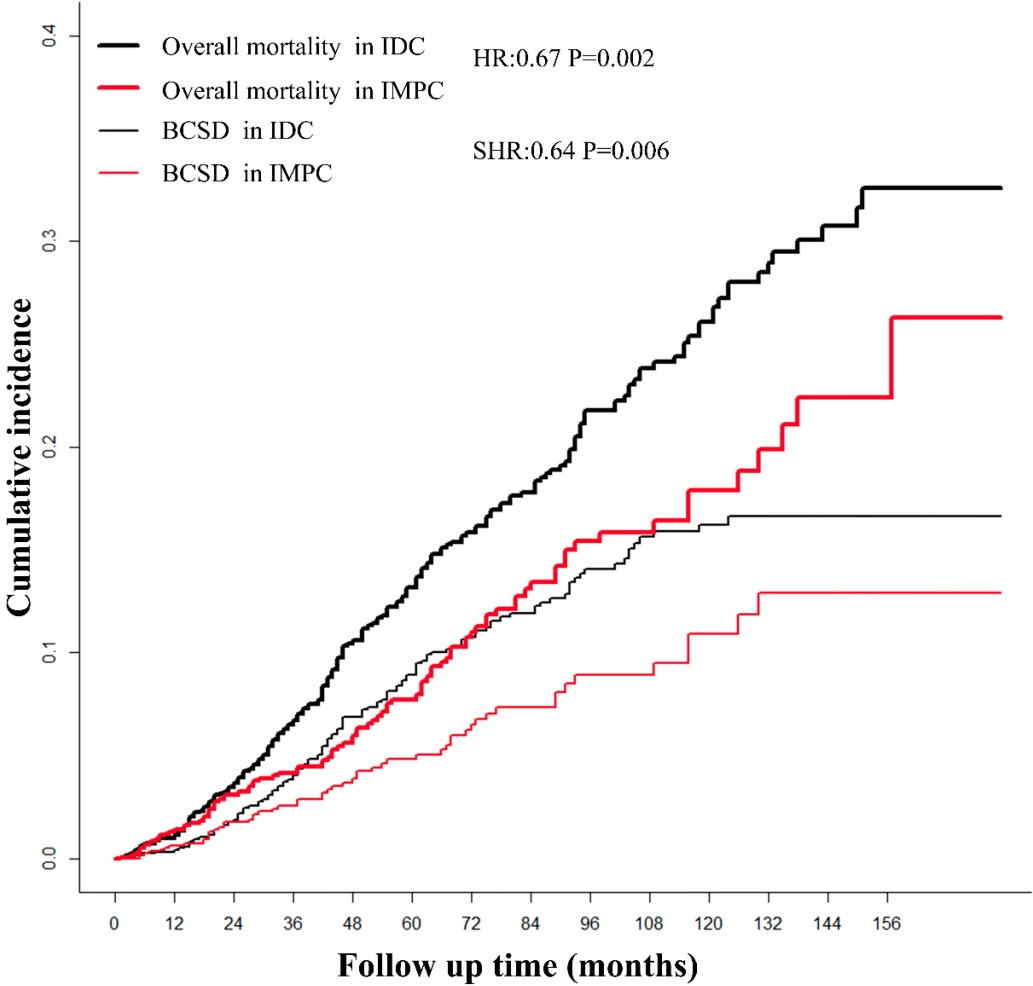
**The survival of patients with IMPC and IDC based on Kaplan-Meier and Gray analysis**. IMPC: Invasive micropapillary carcinoma; IDC: Infiltrative ductal cancer; BCSD: Breast cancer specific death.

The median follow-up time for patients in the study was 64 months (range 1-179 months). After a PSM adjustment, based on Kaplan-Meier analysis, patients with IMPC had a favorable prognosis with marginal statistical significance compared with patients with IDC (overall mortality HR = 0.68; 95%CI, 0.53-0.86; *P* = 0.002). The cumulative incidence of the 3-year, 5-year and 10-year overall mortality for IMPC were 4.17%, 7.74% and 17.89%, respectively, compared with 6.73%, 13.15% and 26.08% respectively, in patients with IDC (**Fig. 1**). Based on Gray method, patients with IMPC had a favorable prognosis with significant statistical significance compared with patients with IDC (BCSD SHR = 0.64; 95%CI, 0.47-0.88; *P* = 0.006). The 3-year, 5-year and 10-year BCSD for the IMPC group was 3.58%, 4.84% and 10.94% compared with 4.05%, 8.93% and 16.24% for the IDC group.

**Figure 2:**
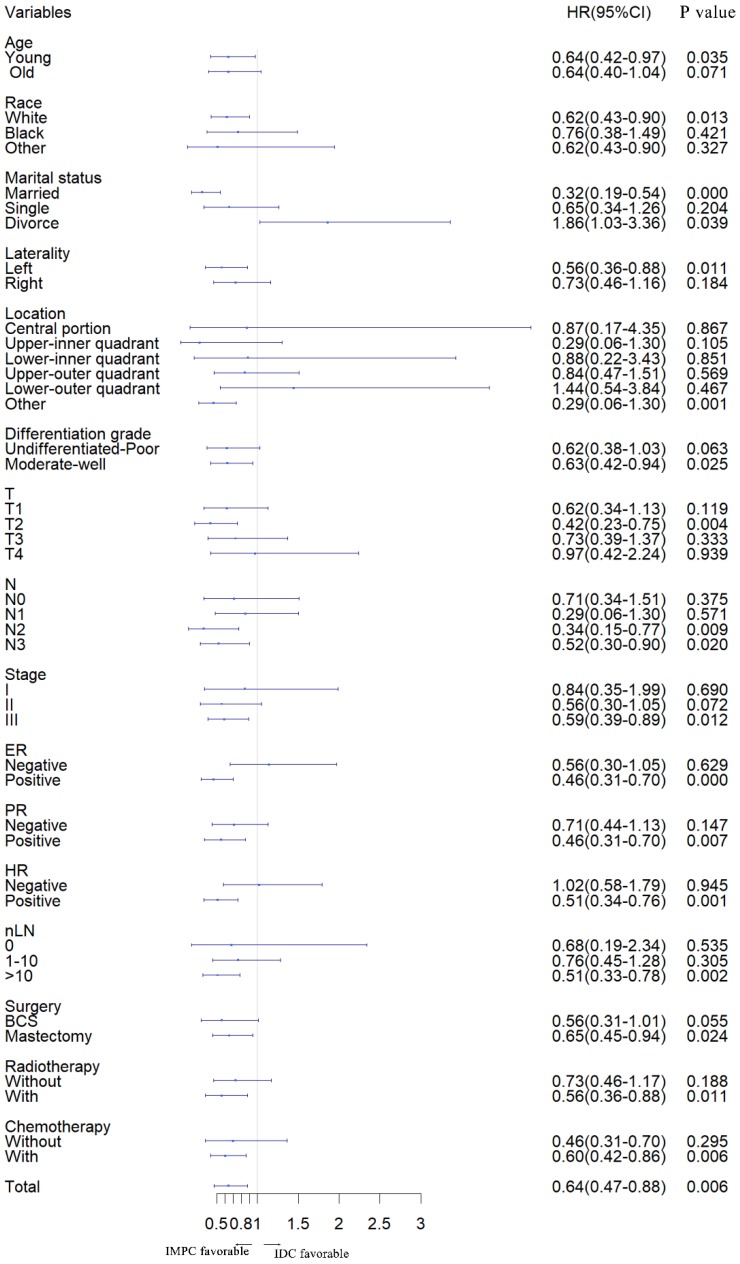
** Forest plot of subgroup analysis.** IMPC: Invasive micropapillary carcinoma; IDC: Infiltrative ductal cancer; SHR: Subdistribution hazard ratio; CI: Confidence index.

Furthermore, we stratified the entire cohort by histology and analyzed BCSD according to patient and tumor characteristics based on univariate analysis of the competing risk regression model. The forest plot of subgroup analysis revealed that, in the young, white, married, left, other location, moderate-well, T2, N2, N3, stage III, ER positive, PR positive, HR positive, number of lymph node resected (nLN) > 10, with radiotherapy, with chemotherapy, with breast conserving and mastectomy surgery subgroups, patients with IMPC had a favorable prognosis compared with patients with IDC. In the divorce subgroup, patients with IDC had a favorable prognosis. Except for listed above subgroups, there was no significant difference between IMPC and IDC subgroup (**Fig. 2**).

### Prognostic factors for IMPC and IDC based on competing risk model

We randomly divided the entire cohort into two parts: a train cohort (249024 patients) and a validation cohort (83009 patients). Relevant baseline variables were similarly distributed in the development and validation cohorts (**Table 2**). The competing risk regression model was used to explore the prognostic factors for patients with IMPC and IDC. Univariate analysis showed the race, marital status, differentiated grade, histology, T-classification, N-classification, stage and ER status, PR status, HR status, nLN, surgery, and chemotherapy were statistically significant prognostic factors for survival. Prognostic factors with a *P* value < 0.05 according to univariate analysis were incorporated into the multivariate analysis. Multivariate analysis showed the race, grade, histology, T-classification, N-classification, HR status, and nLN were statistically significant prognostic factors for BCSD (**Table 3**).

### Construction of a nomogram model

**Figure 3:**
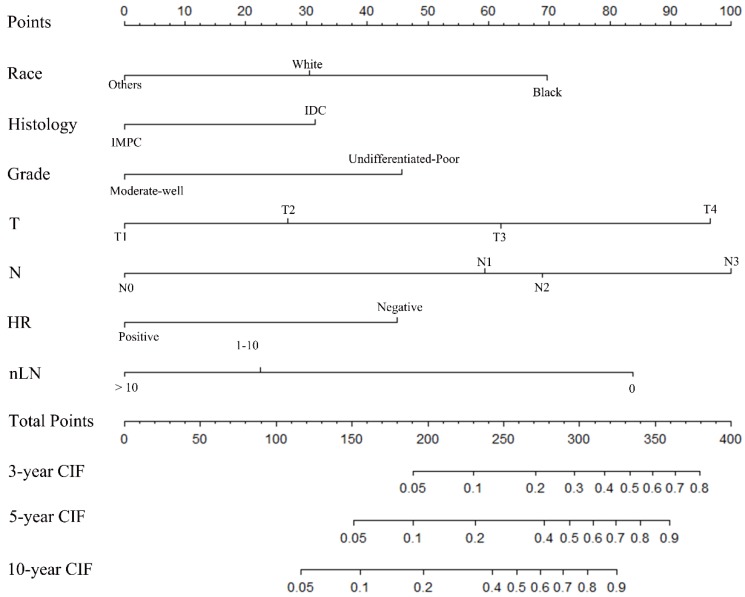
** Nomogram to predict the probability of BCSD.** The factors of marital status, differentiated grade, histology, T-classification, N-classification, HR status, and nLN were included in the model. IMPC: Invasive micropapillary carcinoma; IDC: Infiltrative ductal cancer; BCSD: Breast cancer specific death.

**Figure 4:**
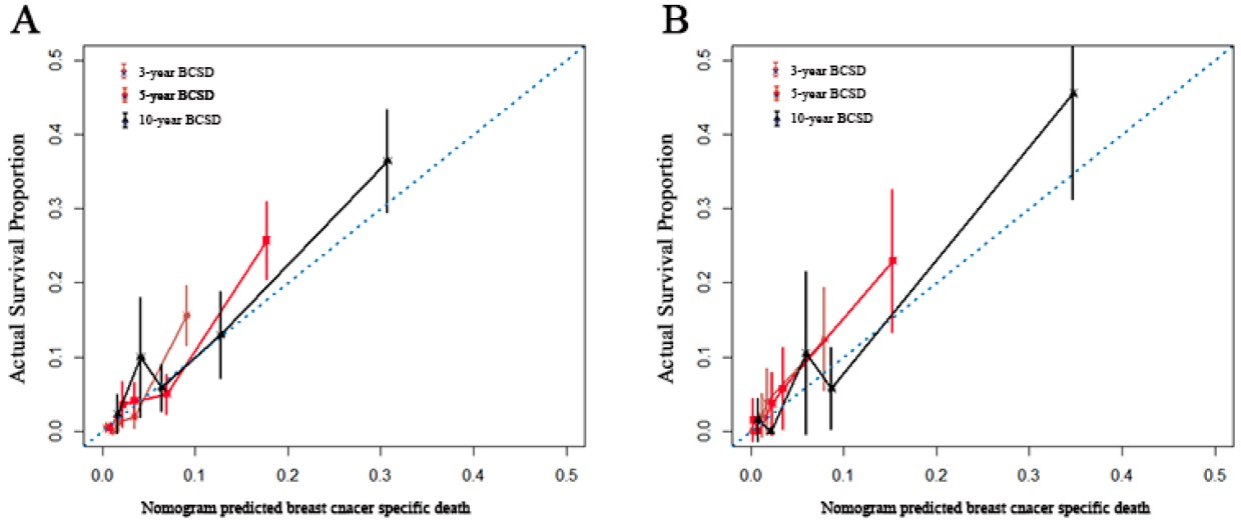
** Internal and external validation of the nomogram.** Calibration curves, which plot the average Gray estimate against the corresponding nomogram for 3-, 5- or 10- year predicted BCSD, were provided to evaluate the performance of the nomogram. The plot presented excellent agreement between the nomogram prediction and the actual observation for 3-, 5 and 10- year CSS. BCSD: Breast cancer specific death.

A nomogram was constructed based on statistically significant factors identified by multivariate analysis from the competing regression model to predict the risk of BCSD in resectable breast cancer. The factors consisted of the race, grade, histology, T-classification, N-classification, HR status, and nLN. N-classification was the most predominant prognostic factor, followed by T-classification, nLN, race, HR status, grade and histology (**Fig. 3**). A vertical line was drawn from the factor to the point scale to determine the score of all variables (detailed in **Table. 4**). We then summarized all of the discrete values and drew a straight line from the total scale to the 3-year, 5-year, and the 10-year BCSD estimated lines to obtain the 3-year, 5-year, and 10-year BCSD rate. The bootstrap method was used to perform the internal validation. The C-index for the nomogram was 0.835 (95%CI: 0.830-0.843). The calibration plots showed a strong agreement for the 3-year, 5-year and 10-year BCSD rate between the nomogram prediction and realistic observation. In the external validation, the C-index for the nomogram was 0.818 (95%CI: 0.813 -0.821). The calibration plots showed a strong agreement for the 3-year, 5-year and 10-year BCSD rate between the nomogram prediction and realistic observation (**Fig. 4**).

### Identification of key genes and pathways in IMPC using bioinformatics analysis

To better understand whether IMPC is a unique biological disease, DEGs were identified between IMPC samples and ICNST samples. In present study, a total of 53 upregulated DEGs and 40 downregulated DEGs of IMPC was identified (**Supplementary** Table 3). Heatmap of the differentially expressed genes between IMPC samples and ICNST samples, with red indicating higher expression and green indicating lower expression (**Supplementary Fig. 2**). The GO analysis results showed that downregulated DEGs were significantly enriched in the following biological processes: extracellular structure organization, serine-type peptidase activity, extracellular matrix, cell-substrate adherens junction, cell-substrate junction. KEGG analysis of selective gene sets shows that upregulated DEGs significantly enriched for processes related to signal pathways: regulation of lipolysis in adipocytes, while downregulated DEGs significantly enriched for processes related to carbon metabolism, Rap1 signaling pathway.

## Discussion

IMPC is a distinct histological subtype of breast cancer with a highly aggressive nature upon initial presentation 21-23. In the current study, IMPC comprised 0.30% of the entire cohort, while previous studies found that IMPC accounted for < 3.0% of all breast cancers 9, 24. Previous studies have indicated that IMPC usually presents with a later stage and has a higher propensity for lymph node involvement 11, 25-27. Consistent with previous studies, our study demonstrated that patients with IMPC had more T3 tumors, a higher rate of N1-N3 nodal metastases, and a higher frequency of ER-positive, PR-positive, and HR-positive status. Previous studies showed that tumors containing the IMPC component tended to present with a larger size. Across clinical presentations, radiographic findings, locations and gross features, there were no obvious differences between IMPC and IDC 28. It remains controversial whether the percentage of the micropapillary component is significant for either lymph node invasion or survival outcomes 1, 29. The criteria for distinguishing between mixed and pure IMPC remain imprecise 28. Molecular-genetic studies found that pure and mixed IMPC were remarkably similar at the genetic level 24. Therefore, the presence of the IMPC component, rather than its percentage should be emphasized. The prognostic significance of tumor size in IMPC patients still requires further validation.

In the current study, IMPC had similar lymph node involvement (52.48%) compared with the range (40.3%-84.8%) of previous studies on IMPC which was significantly higher than that of IDC 8. Ide et al suggested that 8.4% of breast cancer lesions contained the IMPC component in their study of 486 patients. They showed that the presence of the IMPC component alone was a significant predictive factor for lymph node metastasis, even if it was detected in only a small proportion of the tumor 1. Simonetti's study demonstrated that IMPC had high expression of CD24 and low expression of CD44 compared with IDCs, which might explain the increased propensity for lymph node metastasis 30.

The prognosis of IMPC compared with IDC remains a topic of debate. Chen et al analyzed 100 patients with IMPC with a median 5-year follow-up and revealed that IMPC had a worse OS outcome than IDC (59% vs 77%, *P* = 0.004). This was consistent with a retrospective multicenter study by Shi (OS, 75.9% vs 89.5%;* P*<0.001). However, more recently, several studies referring to the outcomes of OS suggested that the OS of IMPC was not inferior to that of IDC 8, 10, 11, 31, 32. Liu et al reported that patients with IMPC had similar prognosis compared with lymph node matched IDC patients, patients with IDC had a favorable prognosis in the T1N2-3 subset, whereas IMPC patients demonstrated comparatively better prognosis in the T2N2-3 subgroup 29. The relationship between IMPC and prognosis may be confused by other factors in the univariate analysis. The prognostic value of IMPC was demonstrated via multivariate analysis (*P* < 0.001). After the adjustment for prognostic factors, especially the T- and N-classification, histology was significantly associated with BCSD (SHR = 0.64; 95%CI, 0.47-0.88; *P* = 0.006). IMPC correlates with an advanced stage (higher T- and N-classification), which is related to an unfavorable survival outcome, and may mask the prognostic value of IMPC for OS in univariate analysis. The established nomogram based on the competing regression model demonstrated that IMPC had favorable clinical outcomes compared with those of IDC, and that prognostic value of IMPC exceeded prognostic factors, grade. Therefore, IMPC might be an independent favorable prognostic factor for patients with breast cancer.

Lymph node metastasis and larger tumor size are widely considered unfavorable prognostic factors in the clinical practice. However, inferior clinical characteristics did not translate into a poor prognosis in the IMPC cohort. Lymph node status, primary tumor size, and clinical characteristics will often determine the follow-up treatment, including chemotherapy, radiation, and endocrine therapy 5. Given the inferior clinicopathologic characteristics, clinicians usually chose aggressive treatment in clinical practice. Patients with IMPC have a higher probability of receiving endocrine therapy compared with patients with IDC due to higher HR positivity. In the study by Tang et al, a total of 898 patients (170 IMPC and 728 IDC) were enrolled. Hormone therapy was administered to 607 patients with HR positivity, of whom 135 (79.41%) were IMPC patients and 472 (64.84%) were IDC patients (*P* < 0.001) 33. Previous studies identified that IMPC patients had higher histological grades than IDC patients (*P* < 0.05) 31, 33, 34. Liu et al observed a significantly higher percentage (92.2%, *P* = 0.044) of IMPC patients receiving a regimen containing anthracycline compared with IDC patients (79.4%) 35. A previous study identified that compared with IDC Patients, IMPC patients received chemotherapy more frequently, although the difference was not statistically significant 31, 33. In addition, patients with IMPC were more likely to undergo resection with axillary lymph node dissection 36. In our study, 38.45% of patients had at least 10 lymph nodes removed in IMPC group, which was significant higher than 30.55% in IDC group (*P* < 0.001).

IMPC as a unique entity is characterized by proliferation of carcinomatous cells organized in nesting pattern, separated from the extracellular matrix by an artifactually created spaces with the cellular apical surface polarity towards the outside^22^, ^37^. Determining whether a specific gene profile may cause abnormalities in IMPC polarity, the present study identified DEGs and signal pathways in IMPC. The GO functions reveal that cell-substrate interactions are among top affected molecular functions in downregulated DEGs for IMPC. This may suggests an important role for downregulated DEGs in promoting cellular polarity and shape maintenance 14, 37. In cell adhesion, cell-cell interactions between cancer cells with endothelium determine the metastatic spread 38. This may explain why IMPC has a higher rate of lymph node metastasis. Unfortunately, the gene expression profiles we used do not provide additional clinical information. Our genomic analyses have not identified direct specific genomic aberration that may explain clinical behavior of IMPC.

To the best of our knowledge, this is the first individual estimated nomogram for IMPC based on a multicentric large-population cohort. The value of the constructed nomogram is its ability to help guide treatment decision making with high cost-efficacy since specific guidelines have not been generated for this rare histological type. The C-index for the nomogram was 0.835 (95%CI: 0.830-0.843), which indicated accurate prognostic prediction for individual survival outcomes. Further studies should focus on how the widely-accepted inferior characteristics of IMPC that did not translate into a worse prognosis could be attributed to more intensive hormone therapy, more frequent chemotherapy, and distinct features, including a higher histological grade or the molecular and genetic mechanisms that underlie the highly aggressive nature of IMPC.

## Limitation

Retrospective analyze are often affected by various biases. Information about is absent in the SEER database, which is important to this study. Thus, there was no clearly defined relationship between the established nomogram and chemotherapy. SEER database also lacks records of molecular information, such as HER2 status and Ki67, such information is also important to establish an intrinsic subtype model.

## Conclusion

In conclusion, our present study focused on IMPC, a rare type of breast cancer that accounted for 0.3% of the entire cohort. IMPC was found to be a favorable independent prognostic factor. The present study identified gene expression profiles and signal pathways of IMPC, which deepen our understanding of the molecular mechanisms of IMPC. The nomogram generated in this study is the first to predict the survival outcomes for IMPC, it accurately predicts early breast cancer outcomes, and provides a reference for informed decision making in clinical practice.

## Supplementary Material

Supplementary figures and tables.

## Figures and Tables

**Table 1:** The characteristics of patients with resectable breast cancer

	Before PSM*			After PSM*		
Risk factors	IDC	IMPC	*P*	IDC	IMPC	*P*
	(n= 330786)	(n= 1247)		(n= 1247)	(n= 1247)	
Age			< 0.001			0.779
Young	188118(56.87%)	638(51.16%)		631(50.6%)	638(51.16%)	
Old	142668(43.13%)	609(48.84%)		616(49.4%)	609(48.84%)	
Race			0.018			0.802
White	262840(79.46%)	951(76.26%)		965(77.39%)	951(76.26%)	
Black	36159(10.93%)	162(12.99%)		154(12.35%)	162(12.99%)	
Other	31787(9.61%)	134(10.75%)		128(10.26%)	134(10.75%)	
Marital status			0.079			0.847
Married	205695(62.18%)	746(59.82%)		759(60.87%)	746(59.82%)	
Single	50051(15.13%)	216(17.32%)		207(16.6%)	216(17.32%)	
Divorce	75040(22.69%)	285(22.85%)		281(22.53%)	285(22.85%)	
Laterality			0.887			0.841
Left	167545(50.65%)	627(50.28%)		622(49.88%)	627(50.28%)	
Right	163196(49.34%)	620(49.72%)		625(50.12%)	620(49.72%)	
Unknown	45(0.01%)	0(0%)		0(0%)	0(0%)	
Location			0.001			0.946
Central portion	15190(4.59%)	64(5.13%)		57(4.57%)	64(5.13%)	
Upper-inner quadrant	41080(12.42%)	176(14.11%)		182(14.6%)	176(14.11%)	
Lower-inner quadrant	19594(5.92%)	84(6.74%)		78(6.26%)	84(6.74%)	
Upper-outer quadrant	119222(36.04%)	372(29.83%)		377(30.23%)	372(29.83%)	
Lower-outer quadrant	24612(7.44%)	105(8.42%)		97(7.78%)	105(8.42%)	
Other	111088(33.58%)	446(35.77%)		456(36.57%)	446(35.77%)	
Differentiation grade			0.039			0.772
Moderate-well	132839(40.16%)	465(37.29%)		472(37.85%)	465(37.29%)	
Undifferentiated-Poor	197947(59.84%)	782(62.71%)		775(62.15%)	782(62.71%)	
T-classification*			< 0.001			0.766
T1	207581(62.75%)	712(57.1%)		718(57.58%)	712(57.1%)	
T2	100128(30.27%)	388(31.11%)		398(31.92%)	388(31.11%)	
T3	15019(4.54%)	109(8.74%)		99(7.94%)	109(8.74%)	
T4	8058(2.44%)	38(3.05%)		32(2.57%)	38(3.05%)	
N-classification *			< 0.001			0.990
N0	219196(66.27%)	605(48.52%)		607(48.68%)	605(48.52%)	
N1	80924(24.46%)	399(32%)		398(31.92%)	399(32%)	
N2	20198(6.11%)	134(10.75%)		137(10.99%)	134(10.75%)	
N3	10468(3.16%)	109(8.74%)		105(8.42%)	109(8.74%)	
Stage *			< 0.001			0.828
I	164286(49.67%)	468(37.53%)		469(37.61%)	468(37.53%)	
II	125302(37.88%)	486(38.97%)		497(39.86%)	486(38.97%)	
III	41198(12.45%)	293(23.5%)		281(22.53%)	293(23.5%)	
ER			< 0.001			0.899
Negative	74369(22.48%)	141(11.31%)		139(11.15%)	141(11.31%)	
Positive	256417(77.52%)	1106(88.69%)		1108(88.85%)	1106(88.69%)	
PR			< 0.001			0.844
Negative	107456(32.49%)	265(21.25%)		261(20.93%)	265(21.25%)	
Positive	223330(67.51%)	982(78.75%)		986(79.07%)	982(78.75%)	
HR			< 0.001			0.898
Negative	69892(21.13%)	135(10.83%)		137(10.99%)	135(10.83%)	
Positive	260894(78.87%)	1112(89.17%)		1110(89.01%)	1112(89.17%)	
nLN			< 0.001			0.773
0	12438(3.76%)	37(2.97%)		38(3.05%)	37(2.97%)	
1-10	220826(66.76%)	772(61.91%)		788(63.19%)	772(61.91%)	
>10	97522(29.48%)	438(35.12%)		421(33.76%)	438(35.12%)	
Surgery			< 0.001			1.000
BCS	202389(61.18%)	699(56.05%)		699(56.05%)	699(56.05%)	
Mastectomy	128397(38.82%)	548(43.95%)		548(43.95%)	548(43.95%)	
Radiotherapy			0.029			0.326
Without	144356(43.64%)	506(40.58%)		482(38.65%)	506(40.58%)	
With	186430(56.36%)	741(59.42%)		765(61.35%)	741(59.42%)	
Chemotherapy			< 0.001			0.658
Without	168950(51.08%)	552(44.27%)		563(45.15%)	552(44.27%)	
With	161836(48.92%)	695(55.73%)		684(54.85%)	695(55.73%)	

*P values obtained from the Chi squared test. *Stage TNM, T, N-classification according to the 8^th^ edition of AJCC staging. Abbreviations: IDC: Infiltrating duct carcinoma. PSM: propensity score matching. IMPC: Invasive micropapillary carcinoma. ER: Estrogen receptor. PR: Progesterone receptor. HR: Hormone receptor. BCS: Breast conserving surgery. nLN: number of lymph node resected.

**Table 2:** Comparisons of patient characteristics of the study population in the train and validation cohorts

	Train cohort			Validation cohort		
Risk factors	IDC	IMPC	*P*	IDC	IMPC	*P*
	(n= 248064)	(n= 960)		(n= 82722)	(n= 287)	
Age			0.002			0.006
Young	140885(57.38%)	497(52.75%)		47233(57.1%)	141(49.13%)	
Old	107179(42.62%)	463(47.25%)		35489(42.9%)	146(50.87%)	
Race			0.012			0.672
White	197182(79.92%)	726(76.44%)		65658(79.37%)	225(78.4%)	
Black	27090(10.79%)	126(13.6%)		9069(10.96%)	36(12.54%)	
Other	23792(9.29%)	108(9.96%)		7995(9.66%)	26(9.06%)	
Marital status			0.109			0.669
Married	154151(62.4%)	574(60.73%)		51544(62.31%)	172(59.93%)	
Single	37505(14.75%)	168(17.23%)		12546(15.17%)	48(16.72%)	
Divorce	56408(22.84%)	218(22.04%)		18632(22.52%)	67(23.34%)	
Laterality			0.537			0.046
Left	125472(50.66%)	502(52.17%)		42073(50.86%)	125(43.55%)	
Right	122558(49.32%)	458(47.83%)		40638(49.13%)	162(56.45%)	
Unknown	34(0.01%)	0(0%)		11(0.01%)	0(0%)	
Location			0.001			0.531
Central portion	11432(4.96%)	47(5.51%)		3758(4.54%)	17(5.92%)	
Upper-inner quadrant	30673(12.16%)	142(13.25%)		10407(12.58%)	34(11.85%)	
Lower-inner quadrant	14653(5.94%)	65(6.33%)		4941(5.97%)	19(6.62%)	
Upper-outer quadrant	89634(35.94%)	282(29.66%)		29588(35.77%)	90(31.36%)	
Lower-outer quadrant	18343(7.32%)	79(8.56%)		6269(7.58%)	26(9.06%)	
Other	83329(33.68%)	345(36.69%)		27759(33.56%)	101(35.19%)	
Differentiation grade			0.023			0.892
Moderate-well	99655(40.74%)	351(39.27%)		33184(40.12%)	114(39.72%)	
Undifferentiated-Poor	148409(59.26%)	609(60.73%)		49538(59.88%)	173(60.28%)	
T-classification*			< 0.001			0.005
T1	155485(62.92%)	545(57.44%)		52096(62.98%)	167(58.19%)	
T2	75235(30.22%)	299(30.95%)		24893(30.09%)	89(31.01%)	
T3	11318(4.39%)	84(8.56%)		3701(4.47%)	25(8.71%)	
T4	6026(2.47%)	32(3.05%)		2032(2.46%)	6(2.09%)	
N-classification *			< 0.001			<0.001
N0	164426(65.77%)	463(46.19%)		54770(66.21%)	142(49.48%)	
N1	60639(24.53%)	308(32.47%)		20285(24.52%)	91(31.71%)	
N2	15120(6.41%)	103(10.79%)		5078(6.14%)	31(10.8%)	
N3	7879(3.29%)	86(10.55%)		2589(3.13%)	23(8.01%)	
Stage *			< 0.001			< 0.001
I	123214(49.36%)	356(35.87%)		41072(49.65%)	112(39.02%)	
II	93990(37.87%)	374(39.86%)		31312(37.85%)	112(39.02%)	
III	30860(12.77%)	230(24.27%)		10338(12.5%)	63(21.95%)	
ER			< 0.001			< 0.001
Negative	55901(23.04%)	111(12.19%)		18468(22.33%)	30(10.45%)	
Positive	192163(76.96%)	849(87.81%)		64254(77.67%)	257(89.55%)	
PR			< 0.001			0.001
Negative	80765(33.19%)	198(24.03%)		26691(32.27%)	67(23.34%)	
Positive	167299(66.81%)	762(75.97%)		56031(67.73%)	220(76.66%)	
HR			<0.001			<0.001
Negative	52510(21.66%)	105(11.61%)		17382(21.01%)	30(10.45%)	
Positive	195554(78.34%)	855(88.39%)		65340(78.99%)	257(89.55%)	
nLN			0.001			0.053
0	9356(3.76%)	31(2.97%)		3082(3.73%)	6(2.09%)	
1-10	165603(66.76%)	592(61.91%)		55223(66.76%)	180(62.72%)	
>10	73105(29.48%)	337(35.12%)		24417(29.52%)	101(35.19%)	
Surgery			<0.001			0.539
BCS	151925(60.66%)	529(54.4%)		50464(61%)	170(59.23%)	
Mastectomy	96139(39.34%)	431(45.6%)		32258(39%)	117(40.77%)	
Radiotherapy			0.303			0.008
Without	108231(44.04%)	403(41.38%)		36125(43.67%)	103(35.89%)	
With	139833(55.96%)	557(58.62%)		46597(56.33%)	184(64.11%)	
Chemotherapy			< 0.001			0.065
Without	126677(50.84%)	421(41.74%)		42273(51.1%)	131(45.64%)	
With	121387(49.16%)	539(58.26%)		40449(48.9%)	156(54.36%)	

*P values obtained from the Chi squared test. *Stage TNM, T, N-classification according to the 8^th^ edition of AJCC staging. Abbreviations: IDC: Infiltrating duct carcinoma. PSM: propensity score matching. IMPC: Invasive micropapillary carcinoma. ER: Estrogen receptor. PR: Progesterone receptor. HR: Hormone receptor. BCS: Breast conserving surgery. nLN: number of lymph node resected.

**Table 3:** Breast cancer-specific death in univariate and multivariate analysis based on competing risk model.

Risk Factors	Univariate analysis^#^		Multivariate analysis^#^	
	SHR (95%CI)	*P*	SHR (95%CI)	*P*
Age				
Young	Ref			
Old	0.8(0.57-1.12)	0.196		
Race				
White	Ref		Ref	
Black	1.99(1.31-3)	0.001	2.06(1.39-3.04)	< 0.001
Other	0.58(0.3-1.15)	0.119	0.59(0.28-1.24)	0.166
Marital status				
Married	Ref			
Single	1.31(0.83-2.06)	0.249		
Divorce	1.46(1.00-2.15)	0.053		
Laterality				
Left	Ref			
Right	1.07(0.77-1.5)	0.685		
Location				
Central portion	Ref			
Upper-inner quadrant	0.49(0.18-1.32)	0.158		
Lower-inner quadrant	0.74(0.25-2.21)	0.591		
Upper-outer quadrant	0.92(0.39-2.16)	0.847		
Lower-outer quadrant	0.73(0.27-2.00)	0.539		
Other	1.20(0.52-2.77)	0.668		
Histology				
IDC	Ref		Ref	
IMPC	0.63(0.44-0.9)	0.012	0.56(0.39-0.81)	0.002
Differentiation grade				
Moderate-well	Ref		Ref	
Undifferentiated-Poor	3.25(2.29-4.61)	< 0.001	2.3(1.54-3.42)	< 0.001
T-classification*				
T1	Ref		Ref	
T2	2.33(1.52-3.57)	< 0.001	1.57(1.00-2.47)	0.049
T3	6.21(3.84-10.04)	< 0.001	2.93(1.62-5.32)	< 0.001
T4	15.35(9.2-25.61)	< 0.001	5.41(2.91-10.06)	< 0.001
N-classification *				
N0	Ref		Ref	
N1	2.88(1.78-4.67)	< 0.001	2.97(1.69-5.21)	< 0.001
N2	4.36(2.49-7.61)	< 0.001	3.54(1.73-7.26)	0.001
N3	9.98(6.03-16.53)	< 0.001	6.38(3.01-13.5)	< 0.001
Stage *				
I	Ref			
II	2.94(1.61-5.37)	< 0.001		
III	10.23(5.78-18.09)	< 0.001		
ER				
Negative	Ref			
Positive	0.29(0.2-0.43)	< 0.001		
PR				
Negative	Ref			
Positive	0.34(0.24-0.48)	< 0.001		
HR				
Negative	Ref		Ref	
Positive	0.31(0.21-0.46)	< 0.001	0.44(0.29-0.68)	< 0.001
nLN				
0	Ref		Ref	
1-10	0.31(0.15-0.64)	0.002	0.27(0.13-0.56)	< 0.001
>10	0.67(0.33-1.39)	0.284	0.17(0.08-0.39)	< 0.001
Surgery				
BCS	Ref		Ref	
Mastectomy	2.81(1.96-4.02)	< 0.001	1.46(0.95-2.25)	0.087
Radiotherapy				
Without	Ref			
With	0.82(0.58-1.14)	0.232		
Chemotherapy				
Without	Ref		Ref	
With	2.24(1.53-3.28)	< 0.001	0.77(0.49-1.2)	0.250

#Univariate and multivariate analyses were conducted using competing risk model. *Stage TNM, T, N-classification according to the 8th edition of AJCC staging. SHR: Subdistribution hazard ratio. HR: Hormone receptor. ER: Estrogen receptor. PR: Progesterone receptor. IDC: Infiltrating duct carcinoma. IMPC: Invasive micropapillary carcinoma. BCS: Breast conserving surgery. nLN: Number of lymph node resected.

**Table 4:** Point assignment and prognostic score in the nomogram

Variable	Risk score
Race	
White	31
Black	70
Other	0
Histology	
IDC	31
IMPC	0
Differentiation grade	
Undifferentiated-Poor	46
Moderate-well	0
T-classification*	
T1	0
T2	27
T3	62
T4	97
N-classification*	
N0	0
N1	59
N2	69
N3	100
HR	
Negative	45
Positive	0
nLN	
0	84
1-10	22
>10	0
Total prognostic score	Estimated 10-year BCSD rate (%)
116	0.05
156	0.10
197	0.20
242	0.40
259	0.50
274	0.60
289	0.70
305	0.80
325	0.90

*Stage TNM, T, N-classification according to the 8^th^ edition of AJCC staging. IDC: Infiltrating duct carcinoma. IMPC: Invasive micropapillary carcinoma. ER: Estrogen receptor. PR: Progesterone receptor. nLN: Number of lymph node resected.
