# Supplementary material for: A Competing Nomogram to Predict Survival Outcomes in Invasive Micropapillary Breast Cancer
Source: J Cancer. 2019 Nov 1;10(27):6801–12. doi: 10.7150/jca.27955 (PMC6909950; doi:10.7150/jca.27955)
Supplement: Supplementary file 1 — Supplementary figures and tables. [file jcav10p6801s1.pdf]

Supplementary Table 1: List of upregulated and downregulated genes in IMPC

gene_id	symbol	gene_name	cytogenetic_location
Downregulation			
100124700	HOTAIR	HOX transcript antisense RNA	12q13.13
100505576	LINC00672	long intergenic non-protein coding RNA 672	17q12
10057	ABCC5	ATP binding cassette subfamily C member 5	3q27.1
100652898	NA	NA	NA
10143	CLEC3A	C-type lectin domain family 3 member A	16q23.1
10155	TRIM28	tripartite motif containing 28	19q13.43
10195	ALG3	ALG3, alpha-1,3- mannosyltransferase	3q27.1
1024	CDK8	cyclin dependent kinase 8	13q12.13
10262	SF3B4	splicing factor 3b subunit 4	1q21.2
10263	CDK2AP2	cyclin dependent kinase 2 associated protein 2	11q13.2
10272	FSTL3	follistatin like 3	19p13.3
10309	CCNO	cyclin O	5q11.2
10322	SMYD5	SMYD family member 5	2p13.2
10370	CITED2	Cbp/p300 interacting transactivator with Glu/Asp rich carboxy-terminal domain 2	6q24.1
10381	TUBB3	tubulin beta 3 class III	16q24.3
10398	MYL9	myosin light chain 9	20q11.23
10409	BASP1	brain abundant membrane attached signal protein 1	5p15.1
10417	SPON2	spondin 2	4p16.3
10460	TACC3	transforming acidic coiled-coil containing protein 3	4p16.3
1048	CEACAM5	carcinoembryonic antigen related cell adhesion molecule 5	19q13.2
10509	SEMA4B	semaphorin 4B	15q26.1
1059	CENPB	centromere protein B	20p13
10615	SPAG5	sperm associated antigen 5	17q11.2
1072	CFL1	cofilin 1	11q13.1
10766	TOB2	transducer of ERBB2, 2	22q13.2
10908	PNPLA6	patatin like phospholipase domain containing 6	19p13.2
10948	STARD3	StAR related lipid transfer domain containing 3	17q12
10954	PDIA5	protein disulfide isomerase family A member 5	3q21.1
10956	OS9	OS9, endoplasmic reticulum lectin	12q13.3-q14.1
10963	STIP1	stress induced phosphoprotein 1	11q13.1
11006	LILRB4	leukocyte immunoglobulin like receptor B4	19q13.42
11082	ESM1	endothelial cell specific molecule 1	5q11.2
11100	HNRNPUL1	heterogeneous nuclear ribonucleoprotein U like 1	19q13.2
11117	EMILIN1	elastin microfibril interfacer 1	2p23.3
11129	CLASRP	CLK4 associating serine/arginine rich protein	19q13.32
11135	CDC42EP1	CDC42 effector protein 1	22q13.1
1116	CHI3L1	chitinase 3 like 1	1q32.1
11226	GALNT6	polypeptide N-acetylgalactosaminyltransferase 6	12q13.13
11230	PRAF2	PRA1 domain family member 2	Xp11.23
11252	PACSIN2	protein kinase C and casein kinase substrate in neurons 2	22q13.2

Upregulation			
100113407	TMEM170B	transmembrane protein 170B	6p24.2
100128822	LINC01003	long intergenic non-protein coding RNA 1003	7q36.1
100131187	TSTD1	thiosulfate sulfurtransferase like domain containing 1	1q23.3
100272228	LINC00894	long intergenic non-protein coding RNA 894	Xq28
100287482	SMKR1	small lysine rich protein 1	7q32.1
100287616	LOXL1-AS1	LOXL1 antisense RNA 1	15q24.1
100287628	NA	NA	NA
100288092	NA	NA	NA
100288152	SLC9A3-AS1	SLC9A3 antisense RNA 1	5p15.33
100289098	GS1-124K5.4	uncharacterized LOC100289098	7q11.21
100289274	DNAJC3-DT	DNAJC3 divergent transcript	13q32.1
100289341	MAN1B1-DT	MAN1B1 divergent transcript	9q34.3
100289635	ZNF605	zinc finger protein 605	12q24.33
100505483	PRKAG2-AS1	PRKAG2 antisense RNA 1	7q36.1
100505761	RPARP-AS1	RPARP antisense RNA 1	10q24.32
100506098	LOC100506098	uncharacterized LOC100506098	7p21.1
100506312	NA	NA	NA
100506409	ELOVL2-AS1	ELOVL2 antisense RNA 1	6p24.2
100506492	DSCAM-AS1	DSCAM antisense RNA 1	21q22.2
100506686	IQCH-AS1	IQCH antisense RNA 1	15q23
100506948	NA	NA	NA
100507015	NA	NA	NA
100507213	NA	NA	NA
100507316	MINCR	MYC-induced long non-coding RNA	8q24.3
100507399	NA	NA	NA
100652765	NA	NA	NA
10098	TSPAN5	tetraspanin 5	4q23
10125	RASGRP1	RAS guanyl releasing protein 1	15q14
10189	ALYREF	Aly/REF export factor	17q25.3
10202	DHRS2	dehydrogenase/reductase 2	14q11.2
10205	MPZL2	myelin protein zero like 2	11q23.3
10207	PATJ	PATJ, crumbs cell polarity complex component	1p31.3
10267	RAMP1	receptor activity modifying protein 1	2q37.3
10268	RAMP3	receptor activity modifying protein 3	7p13
10325	RRAGB	Ras related GTP binding B	Xp11.21
10390	CEPT1	choline/ethanolamine phosphotransferase 1	1p13.3
10420	TESK2	testis associated actin remodelling kinase 2	1p34.1
10434	LYPLA1	lysophospholipase I	8q11.23
10512	SEMA3C	semaphorin 3C	7q21.11
10560	SLC19A2	solute carrier family 19 member 2	1q24.2
10565	ARFGEF1	ADP ribosylation factor guanine nucleotide exchange factor 1	8q13.2
10742	RAI2	retinoic acid induced 2	Xp22.13
10793	ZNF273	zinc finger protein 273	7q11.21

10873	ME3	malic enzyme 3	11q14.2
10884	MRPS30	mitochondrial ribosomal protein S30	5p12
10923	SUB1	SUB1 homolog, transcriptional regulator	5p13.3
1101	CHAD	chondroadherin	17q21.33
11059	WWP1	WW domain containing E3 ubiquitin protein ligase 1	8q21.3
11112	HIBADH	3-hydroxyisobutyrate dehydrogenase	7p15.2
11122	PTPRT	protein tyrosine phosphatase, receptor type T	20q12-q13.11
11162	NUDT6	nudix hydrolase 6	4q28.1
100506687	NA	NA	NA
100506802	NA	NA	NA

Supplementary table 2. Gene ontology analysis of downregulated differentially expressed genes associated with IMPC

	ID	Description	Count	p.adjust
	GO:0043062	extracellular structure organization	44	1.43E-16
	GO:0030198	extracellular matrix organization	41	1.43E-16
	GO:0050900	leukocyte migration	44	6.33E-14
	GO:0032963	collagen metabolic process	19	1.19E-08
	GO:0030574	collagen catabolic process	14	8.98E-08
	GO:0002455	humoral immune response mediated by circulating immunoglobulin	18	3.80E-07
	GO:0030449	regulation of complement activation	16	3.80E-07
	GO:2000257	regulation of protein activation cascade	16	3.80E-07
	GO:0002920	regulation of humoral immune response	17	3.80E-07
	GO:0016064	immunoglobulin mediated immune response	21	3.80E-07
	GO:0022617	extracellular matrix disassembly	15	3.80E-07
	GO:0019724	B cell mediated immunity	21	3.80E-07
	GO:0006958	complement activation, classical pathway	17	4.52E-07
	GO:0006898	receptor-mediated endocytosis	27	4.52E-07
	GO:0002449	lymphocyte mediated immunity	26	7.17E-07
BP	GO:0006956	complement activation	18	1.64E-06
	GO:0002460	adaptive immune response based on somatic recombination of immune receptors built from immunoglobulin superfamily domains	25	2.70E-06
	GO:0002673	regulation of acute inflammatory response	17	3.47E-06
	GO:0007409	axonogenesis	30	6.37E-06
	GO:0002697	regulation of immune effector process	28	6.37E-06
	GO:0030335	positive regulation of cell migration	31	6.86E-06
	GO:0030199	collagen fibril organization	10	7.47E-06

	GO:0050727	regulation of inflammatory response	28	7.58E-06
	GO:0072376	protein activation cascade	18	9.21E-06
	GO:0070613	regulation of protein processing	17	1.38E-05
	GO:1903317	regulation of protein maturation	17	1.58E-05
	GO:0061564	axon development	30	3.01E-05
	GO:0061138	morphogenesis of a branching epithelium	17	5.45E-05
	GO:0016485	protein processing	23	6.61E-05
	GO:0006909	phagocytosis	23	6.72E-05
	GO:0060485	mesenchyme development	20	6.72E-05
CC	GO:0031012	extracellular matrix	46	2.72E-16
	GO:0005578	proteinaceous extracellular matrix	36	1.15E-12
	GO:0005925	focal adhesion	32	2.10E-09
	GO:0005924	cell-substrate adherens junction	32	2.10E-09
	GO:0030055	cell-substrate junction	32	2.33E-09
	GO:0044420	extracellular matrix component	14	7.97E-06
	GO:0005788	endoplasmic reticulum lumen	21	3.67E-05
	GO:0009897	external side of plasma membrane	20	7.51E-05
MF	GO:0008236	serine-type peptidase activity	23	3.89E-06
	GO:0017171	serine hydrolase activity	23	3.89E-06
	GO:0003823	antigen binding	19	3.89E-06
	GO:0004252	serine-type endopeptidase activity	21	6.04E-06

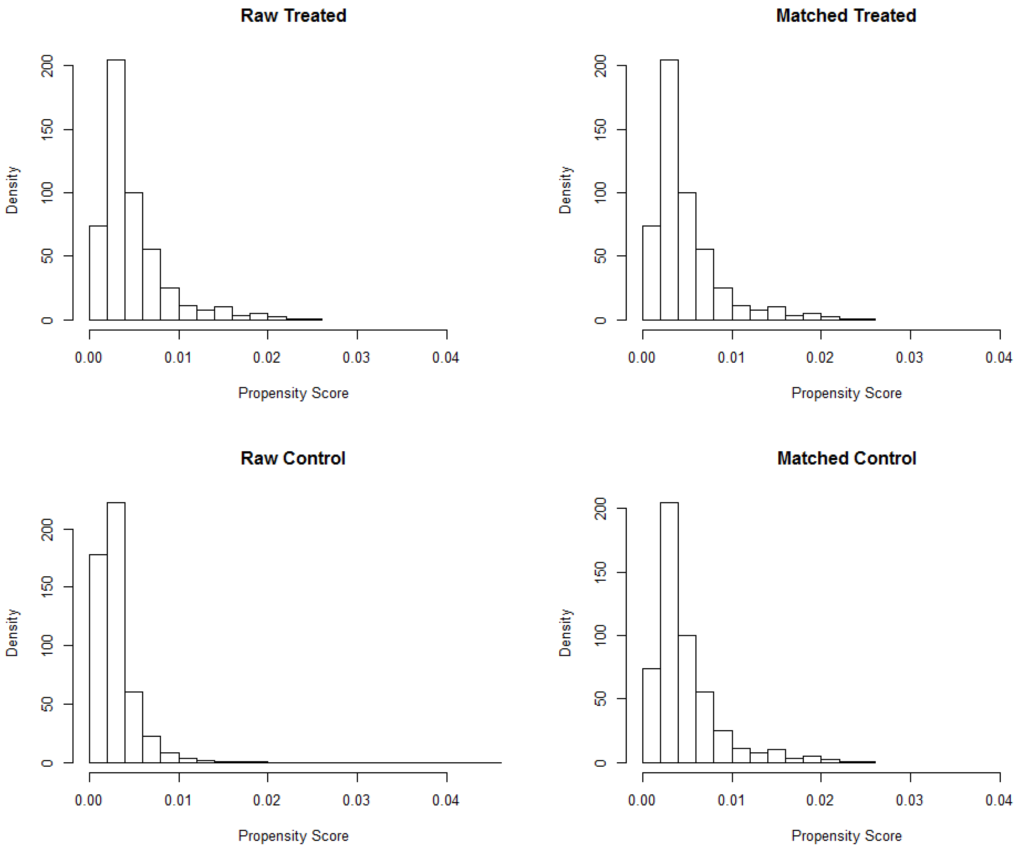

Supplementary Figure 1: **Histograms of the propensity scores before and after matching**.

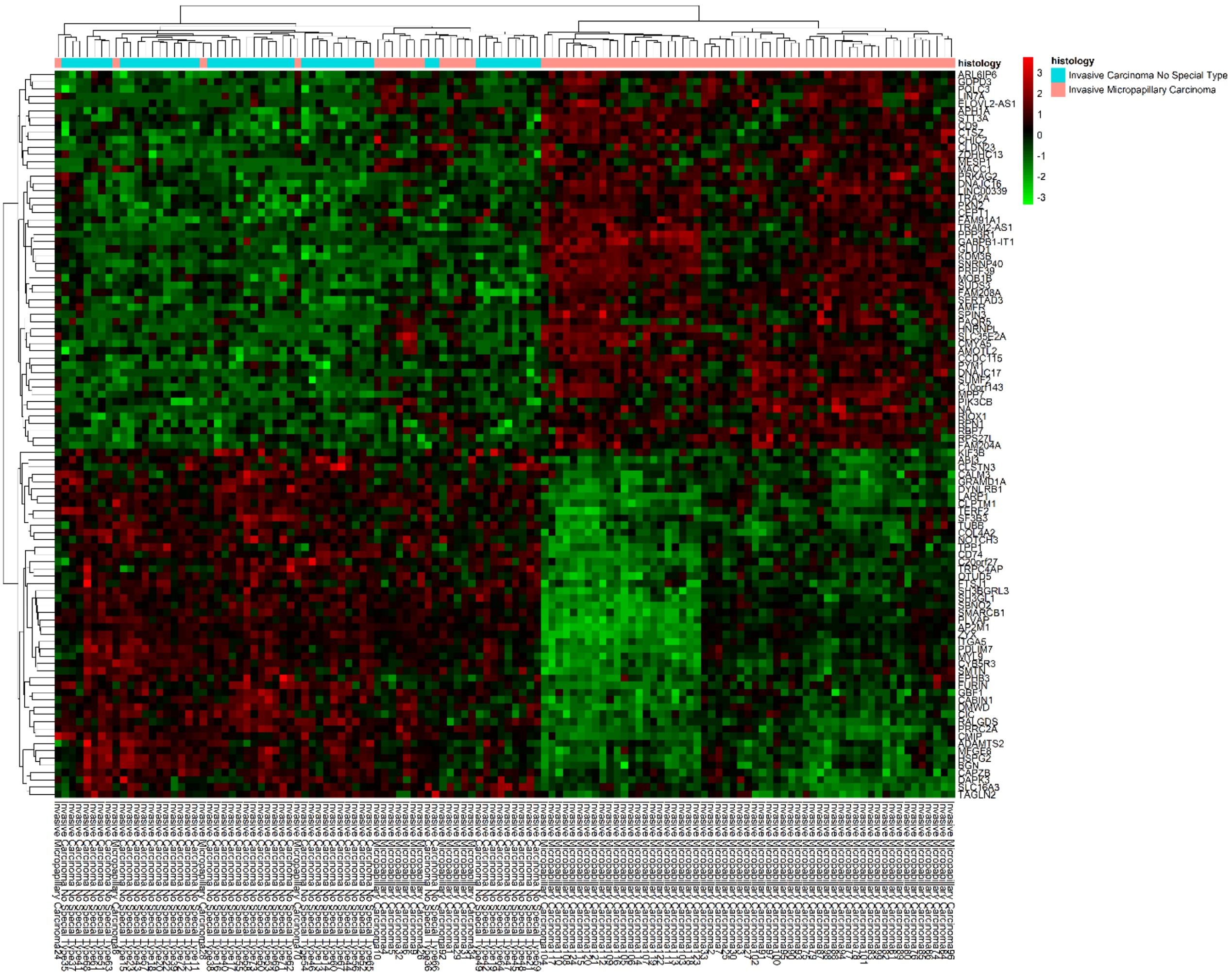

Supplementary Figure 2: Heatmap of the differentially expressed genes between IMPC samples and ICNT samples, with red indicating higher expression and green indicating lower expression.
